# Cryfibrinogen-Associated Glomerulonephritis and Monoclonal Gammopathy of Renal Significance—Case Report and Literature Review

**DOI:** 10.3390/jcm14051656

**Published:** 2025-02-28

**Authors:** Edoardo Terzolo, Laura Solfietti, Michela Ferro, Antonella Barreca, Massimo Milan, Roberta Fenoglio, Savino Sciascia, Dario Roccatello

**Affiliations:** 1 University Center of Excellence on Nephrologic, Rheumatologic and Rare Diseases (ERK-Net, ERN-Reconnect and RITA-ERN Member) with Nephrology and Dialysis Unit and Center of Immuno-Rheumatology and Rare Diseases (CMID), Coordinating Center of the Interregional Network for Rare Diseases of Piedmont and Aosta Valley, San Giovanni Bosco Hub Hospital ASL Città di Torino and Department of Clinical and Biological Sciences, 10154 Turin, Italy; edoardo.terzolo@gmail.com (E.T.); roberta.fenoglio@unito.it (R.F.); dario.roccatello@unito.it (D.R.); 2 Division of Pathology, Città Della Salute e Della Scienza Hospital, University of Turin, 10154 Turin, Italy; 3 Trasfusional Medicine, San Giovanni Bosco Hub Hospital ASL Città di Torino, 10154 Turin, Italy

**Keywords:** cryoprotein, cryofibrinogen, membranoproliferative glomerulonephritis, MPGN, MGUS, fibrinogen kidney disease, kidney biopsy

## Abstract

**Background/Objectives**: Cryofibrinogenemia, characterized by plasma cryoprecipitation of fibrinogen and related proteins, is a rare and often under-recognized entity that can present with significant renal involvement. **Methods**: we describe a 66-year-old woman with progressive renal failure due to membranoproliferative glomerulonephritis driven by cryofibrinogen deposits. Her clinical course was marked by relapsing–remitting disease with limited response to high-dose corticosteroids but significant improvement following plasma exchange. Over seven years, she underwent three kidney biopsies, revealing progressive histopathological changes, including glomerular cryofibrinogen deposits and evolving chronicity. A detailed review of the literature identified 50 cases of cryofibrinogenemia, highlighting its association with monoclonal gammopathies, malignancies, and autoimmune diseases. **Results**: our case uniquely underscores the pathogenic interplay between cryofibrinogenemia and a monoclonal IgG-kappa paraprotein, which was found to directly stabilize fibrinogen and drive cryoprecipitation. This novel observation aligns cryofibrinogenemia with monoclonal gammopathy of renal significance, expanding the diagnostic and therapeutic landscape for this entity. **Conclusions**: this report also highlights the pivotal role of kidney biopsy with electron microscopy in diagnosing cryofibrinogen-associated renal disease, particularly when conventional biomarkers are insufficient. Moreover, our findings emphasize the therapeutic utility of plasmapheresis and the potential need for therapies aimed at eliminating the pathogenetic monoclonal antibody in managing refractory cases. Enhanced awareness and further research into this rare entity are essential for advancing patient care and outcomes.

## 1. Introduction

Cryofibrinogen is a cryoprotein first described by Korst et al. in 1955 in a patient with a pulmonary neoplasm associated with migratory thrombophlebitis [1]. This abnormal cryoprotein is distinguished from its more common counterpart, cryoglobulins, as it can only be detected in plasma cryoprecipitate and not in serum.

This abnormal cryoprotein is distinguished from its more common counterpart, cryoglobulins, as it can only be detected in plasma cryoprecipitate and not in serum. The cryoprecipitates in cryofibrinogenemia consist of fibrinogen, fibrin, fibronectin, and smaller amounts of other proteins [2,3]. It may or may not be associated with concurrent cryoglobulinemia. Aside from the demonstration of circulating cryofibrinogenemia, no other specific biomarkers are known; diagnosis is made through an integration of clinical, laboratory, and histological evidence of organ damage related to the circulating cryoprotein.

In patients with symptoms related to cryoproteinemia the prevalence of circulating cryofibrinogen is reported to be elevated [4,5]. In the inpatient population, the prevalence has been reported to be as high as 3% [6]. On the other hand, the prevalence of cryofibrinogenemia in the healthy population has been reported to be high as well [7]. The possibility that the quality of the pre-analytical procedures used can lead to a substantial overestimation of these evaluations cannot be excluded. Indeed, the definition of cryofibrinogen as a cryoprecipitate that develops following plasma refrigeration, but does not occur in cold serum [8], is by itself misleading. If this was the case, several plasma specimens potentially contain cryobibrinogen, at least in trace amounts. Instead, cryofibrinogen is one of the cryoproteins that precipitates in plasma maintained at 4–6 Celsius degrees for over 72 h, and dissolves again at room temperature.

Cryofibrinogen is not detected in the serum, but only in the plasma, which allows it to be distinguishable from cryoglobulin. Assuming the correct collection, preservation, and processing of the specimen, cryofibrinogenemia should be defined as a pathological condition when promoting the development of clinical manifestations related to the obstruction of the microvasculature by cryoprecipitable proteins, especially if supported by histopathological evidence.

Cryofibrinogenemia can be classified into two main forms: primary (essential) or secondary to an underlying condition, such as an immunological disease, infection, or cancer [7]. The clinical features of the disease vary widely, with skin manifestations (such as livedo, purpura, ulcers, and necrotic lesions) being the most commonly reported. Arthralgia and constitutional symptoms are also frequently described, and the peripheral nervous system may be affected, typically presenting as mononeuritis multiplex. Vascular thrombosis, which is most evident in the cutaneous findings associated with the disease, is considered a hallmark of cryopathies and is the most common pathological association with cryofibrinogenemia.

In the presence of circulating cryofibrinogenemia, kidney involvement is rare and scarcely documented in the literature. It most commonly presents with a membranoproliferative pattern of injury, immunofluorescence positivity for fibrinogen, and distinctive large microtubular structures on electron microscopy [8]. While validated diagnostic criteria are lacking due to the rarity of the disease, integrating clinical, laboratory, and histological data can help clinicians establish a link between organ damage and this cryopathy.

Thus, cryofibrinogenemia may represent an under-recognized cause and pathogenic factor in certain forms of kidney disease.

## 2. Methods

We conducted a literature review using PubMed with the search query ‘cryofibrinogenemia OR cryofibrinogenaemia OR cryofibrinogen’, focusing on case reports and series published in English since 2000, where individual patient clinical and laboratory data were available for further analysis. Case reports or series without accessible individual patient data, as well as studies involving patients under 18 years of age, were excluded.

## 3. Case Description

We present the case of a 66-year-old woman with rapidly progressive renal failure due to membranoproliferative glomerulonephritis associated with cryofibrinogen deposits. Initial treatment with iv 1 g methylprednisolone pulses resulted in a poor response, and plasma exchange (PE) was started. This intervention stabilized her clinical and serological status for several years. Her chronic kidney disease subsequently progressed over the years and became less responsive to therapeutic interventions. Over the course of her illness, the patient underwent three kidney biopsies, which coincided with disease flares (2017–2018–2023), and documented a pathogenetically unexpectable evolution of the disease. At the age of 62 years old, she presented with impaired kidney function (sCr 1.34 mg/dL—eGFR CKD-EPI 45 mL/min) in February 2017, which progressively declined in the first year of follow-up (sCr 2.1 mg/dL) and was associated with significant 24 H urinary protein excretion (24 h-UPE) of 3.7 g and microhematuria.

The patient’s family and work history were unremarkable.

Medical history was positive for obesity (BMI 32), systolic hypertension under treatment since 2016 with a beta-blocker, hives responsive to antihistamines, and a prior hospitalization for acute pancreatitis 10 years prior. IgG-kappa monoclonal gammopathy was reported but was not confirmed in subsequent determinations.

Autoimmunity screening (ANAs, ENA, anti-dsDNA) was negative; the C3 and C4 levels were within normal range. HCV/HBV/HIV infections were ruled out. No other organ dysfunction or cutaneous findings were noted.

**Figure 1:**
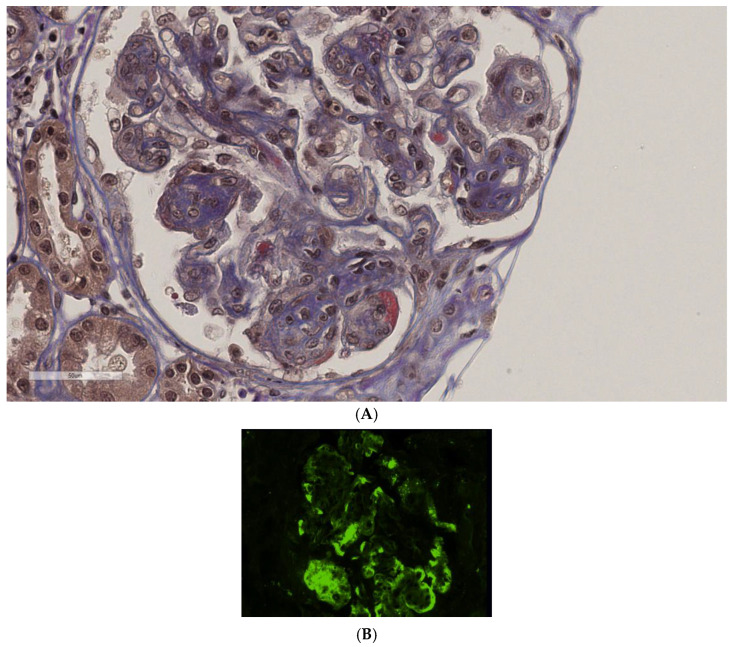

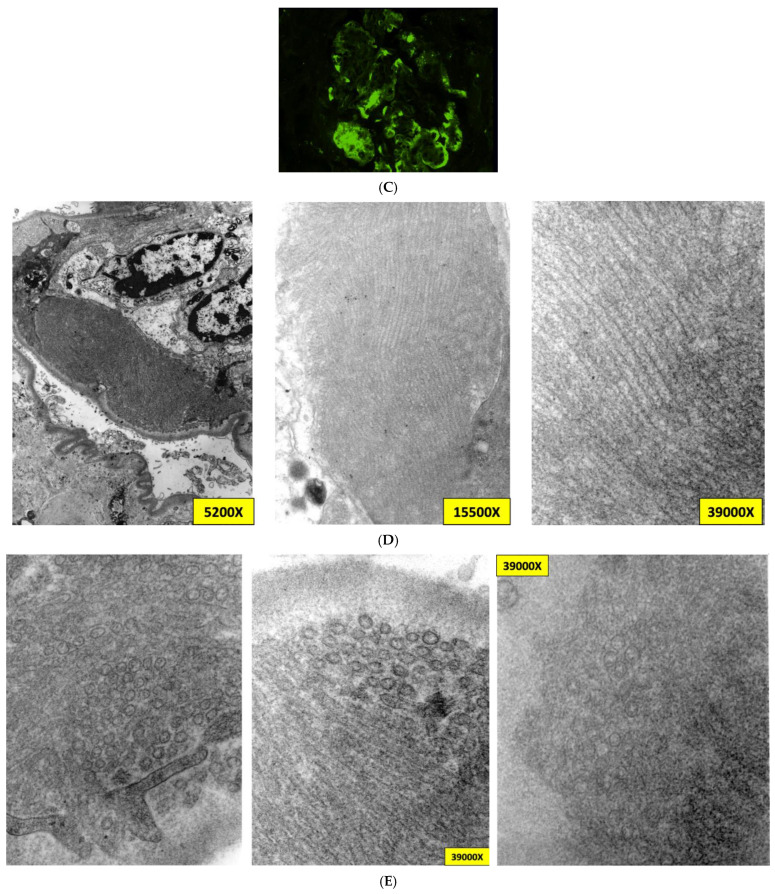
(**A**) Glomerulus exhibiting membrano-proliferative pattern of injury in AFOG coloration; (**B**) immunofluorescence for C3; (**C**) immunofluorescence for fibrinogen; (**D**) subendothelial deposits with a structured appearance, the formation of microtubules, and annular structures; (**E**) microtubules with central bore and annular structures at higher magnification.

A kidney biopsy was performed (Figure 1A–E). The cortical tissue included 42 glomeruli. Two of them had ischemic global sclerosis; two others presented with globally collapsed and folded basement membranes. Three glomeruli presented areas (5–25% of the tuft) in which segmental adhesions of sclerosis and hyalinosis were present. Two glomeruli exhibited fibrinoid necrosis. The remaining glomeruli had free urinary spaces and showed diffuse mesangial expansion, mainly due to extracellular matrix accumulation and, to a lesser amount, to mesangial hypercellularity. Glomerular basement membranes were slightly expanded in width (Figure 1A). In many segments, capillary lumens were poorly visible due to the presence of polymorphonucleate cells, mononucleate cells, and mesangial expansion. In PTAH (phosphotungstic acid-haematoxylin stain)- and AFOG (Acid Fuchsin Orange G)-stained sections, subendothelial and mesangial deposits of various sizes were present. The interstitium, composed of finely fibrous connective tissue, contained some tubules with endoluminal calcium salt deposits. Sporadic tubules exhibited isometric vacuolization of the cytoplasm of epithelial cells. Some foam cells could be sporadically detected. Immunohistochemical investigation highlighted generalized and diffuse (occasionally in granules) positivity for C3 (+++) (Figure 1B), positivity for fibrinogen, lower positivity for C1q (+), and light positivity for IgM (−/+); kappa and lambda chains were also present. C3 positivity (+++) was also observed in some tubules. An ultrastructural examination demonstrated the presence of structured microtubular deposits (45–80 nm) and annular structures (80–100 nm) with double contours (Figure 1D,E). Numerous tubular structures presented a prominent central cylinder that sometimes showed partial double lamellation.

In conclusion, optical microscopy was suggestive of a membrano-proliferative pattern of injury, immunofluorescence was positive for C3 and fibrinogen, and an ultrastructural examination highlighted the presence of typical organized deposits. A diagnosis of cryofibrinogen-associated glomerulonephritis was made. Cryoglobulin determination was negative, while a cryoprecipitate from plasma in EDTA (4%) was detected.

The patient was given 1 g × 3 iv methylprednisolone over three consecutive days, followed by 1 mg/kg/die prednisone per os. However, kidney function continued to worsen rapidly (sCr 5.6 mg/dL—eGFR 8 mL/min), with proteinuria of 1.2 g/24 h. Consultation from the Reference Center for Rare Diseases was sought, and in February 2018, the patient was admitted to the Nephrology ward of Giovanni Bosco Hospital, Turin. The monoclonal component previously found at low titer levels was no longer detectable. Considering the need to rapidly remove the potentially pathogenic circulating cryofibrinogen, and its characteristics (being a mix of molecules with a high kDa and long half-life), eight plasma exchange (PE) sessions were carried out [Total Plasma Volume: 2000 cc, reinfusion with NaCl 0.9% + albumin 5%]. Following this treatment, plasma cryoprecipitate was no longer detectable, renal function rapidly improved (sCr 2.5 mg/dL—eGFR 21 mL/min—March 2018), and proteinuria decreased (24 h-UPE <1 g).

**Figure 2:**
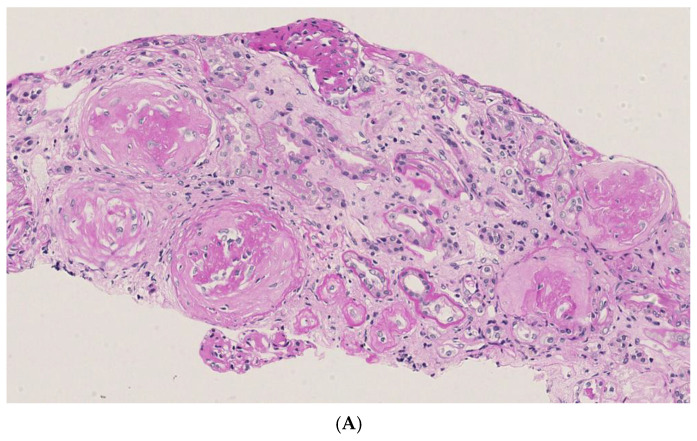

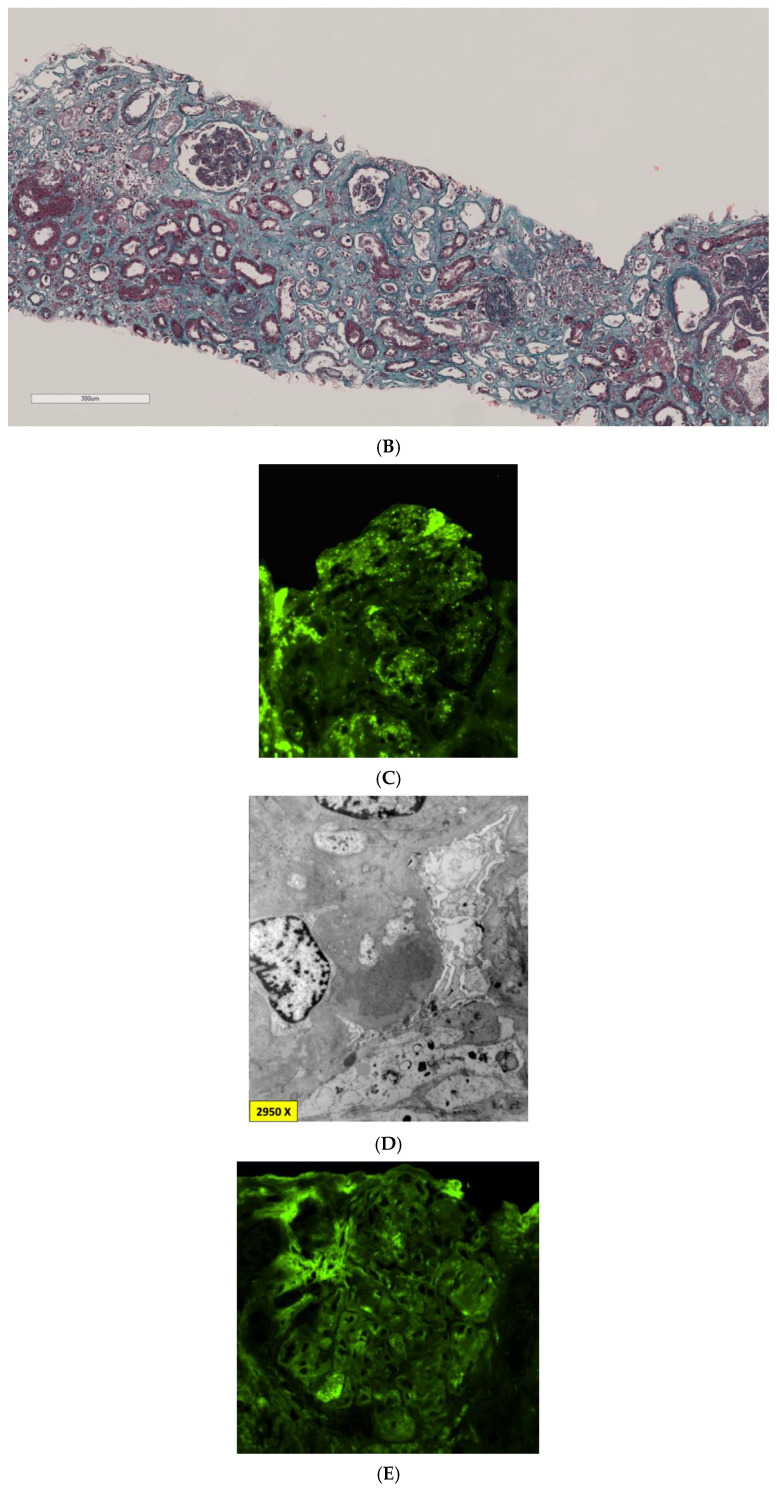
(**A**) HE staining: glomerular sclerosis and interstitial fibrosis increased; (**B**) trichrome staining showing glomerular sclerosis and interstitial fibrosis increasing; (**C**) immunofluorescence for C3 (+); (**D**) ultrastructural examination of a deposit; (**E**) immunofluorescence for fibrinogen (+).

Plasma cryoprecipitate was detected again (3%) five months later (August 2018), and a second kidney biopsy was then performed (Figure 2A–E). A total of 30 glomeruli were examined, 10 of which showed global ischemic-type sclerosis of the tuft. Two glomeruli showed segmental areas of sclerosis/sclero-jalinosis affecting 20% of the capillary loops. The remaining glomeruli showed free urinary spaces with moderately expanded mesangial axes, mainly due to an increase in the mesangial matrix and only occasionally due to an increase in the cellular component. In the PTAH and AFOG stains, occasional subendothelial deposits in the basal membranes were evident. The interstitium, consisting of finely fibrous connective tissue, was moderately expanded in some areas. The immunohistochemical investigation showed mild positivity for C3 (+) in rare fine granules scattered in the tuft, and for fibrinogen (+), was observed to be focal and segmental in small areas of the tuft. Ultrastructural examination demonstrated the presence of microtubular deposits. In conclusion, compared to the previous examination, there was an increase in nodular mesangial expansion, with a reduction in immune deposits and endocapillary proliferation. Given the persistence of cryofibrinogen deposits, albeit to a lesser extent compared to the previous biopsy, two PE sessions were carried out, despite the high chronicity burden and limited endocapillary proliferation.

In the following years (September 2018–January 2022), renal function remained stable (sCr 1.5–1.7 mg/dL) with non-significant proteinuria (<0.5 g/24 h) and no detectable plasma cryoprecipitate. In February 2022, the patient had mild flu-like symptoms and tested positive for SARS-CoV2. After 2 months, plasma cryoprecipitate was once again detectable (4%), and soon after proteinuria increased (24 h UPE 3.4 g) and kidney function declined (sCr 2.7 mg/dL). Given the efficacy of steroid treatment on proteinuria, two sessions of plasmapheresis combined with iv hydrocortisone 500 mg (one bolus after each session) were performed. At discharge, sCr was 2.5 mg/dL with proteinuria of 1.8 g/day and a plasma cryoprecipitate of 3%. Kidney function remained stable for a few months. In January 2023, two sessions of plasmapheresis and two pulses of hydrocortisone 500 mg IV were administered. In January 2023, the patient exhibited, for the first time, a frank nephrotic syndrome (24 h UPE 9 g/24 h; serum protein 55 g/L), with worsening of renal function (sCr 3 mg/dL). Plasma cryocroprecitate also increased by up to 4%. She was therefore readmitted to the Nephrology ward and a third renal biopsy was undertaken.

Seventeen glomeruli were examined: seven (41%) showed global sclerosis of the tuft, while the remaining ten glomeruli showed moderately expanded mesangial axes due to an increase in both the cellular component and mesangial matrix, which was sometimes predominant. In occasional segments, the fundamental substance appeared nodular, with the cellular component being poorly represented. The basal membranes were diffusely thickened with features of duplication.

Subendothelial deposits in the basal membranes were evident in PTAH- and AFOG-stained sections. The interstitium, consisting of finely fibrous connective tissue, was moderately expanded in some areas and included some atrophic tubules (trichomic staining).

An immunohistochemical investigation performed on material fixed with anti-C4d antibodies was positive (++), with a diffuse but irregular distribution in some segments of the glomerular basal membrane and mesangial axes.

A moderate positivity for IgG (++) and C1q (+/++), and mild positivity for C3 (+), in rare fine granules located in limited segments of the basal membrane in the intramembranous and subendothelial areas, and in occasional mesangial axes, was observed. Immunofluorescence with anti-light chain kappa and lambda antibodies (tested with two different clones) showed moderate positivity for kappa light chains (++), with a pattern similar to that of IgG, while lambda chains were negative. Antibodies directed against IgG subclasses (IgG1, IgG2, IgG3, IgG4) tested positive for IgG3 (++) only.

In conclusion, contrasts with previous biopsies IgG–kappa deposits are highlighted; chronic injury accrues in the context of membrano-proliferative glomerulonephritis.

Meanwhile, a further worsening of renal function (sCr 5 mg/dL) and a 24 h-UPE of 4.6 g with rising cryofibrinogen levels (7%) were observed. Three more PE sessions were carried out, with iv 500 mg hydrocortisone administered after each session, which led to the rapid tapering of deflazacort per os. Renal function slightly improved (sCr 3–4 mg/dL); cryofibrinogen decreased but was still detectable (4%). Trying to avoid further progression and the need for renal replacement therapy, an intensified PE regimen was performed in September 2023. A total of seven sessions were carried out, associated with hydrocortisone 500 mg × 3 iv followed by deflazacort with complete taper over 8 weeks. At the withdrawal of steroid therapy, no cryofibrinogen was detectable; sCr was 3.4 mg/dL and 24-UPE was 2.3 g.

In December 2023, the patient presented with acute kidney injury (sCr 10 mg/dL, urea 305 mg/dL) and oliguria. Cryofibrinogen was once again present and quantified at 7%. Seven plasmapheresis sessions were again carried out, with steroid treatment with 500 mg × 3 methylprednisolone followed by deflazacort per os. Hemodialysis was performed for fluid overload management and the reversal of uremic symptoms. Urine output rapidly improved after the start of PE. In May 2024, the patient presented with anuria, pitting edema and dyspnea. Cryofibrinogenemia was once again detectable and quantified at 5%. Hemodialysis, PE, and the same steroid cycle as before were carried out. Anuria once again abated, but due to persistent uremic symptoms, the patient was started on a once-weekly dialysis schedule. The patient was started on daratumumab, an anti-CD38 monoclonal antibody, based on the data of the last biopsy, which highlighted IgG3 kappa monoclonal deposits, and laboratory evidence suggesting that the serum monoclonal gammopathy could stabilize cryofibrinogen (see ‘*Literature Review*’).

The patient is currently continuing follow-up in our Center and is currently on an incremental dialysis protocol.

## 4. Results of the Literature Review

**Figure 3:**
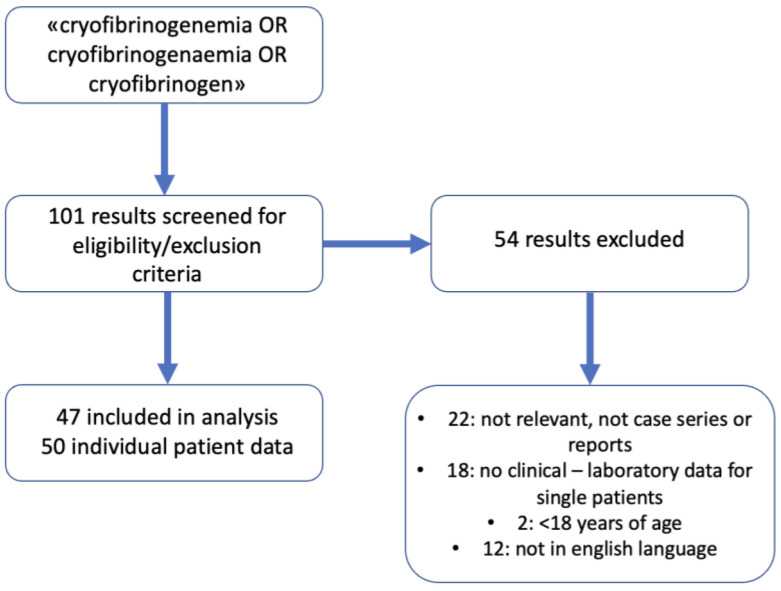
Search strategy.

Forty-seven papers were included, with a total of 50 patients. The most common of relevant comorbidities included malignancies, autoimmune conditions, and monoclonal gammopathies. A minority of the cases reviewed (15/50) were categorized as primary. Over 50% of the secondary cases (19/35) were associated with malignancies, most of which were hematological (11/19). Five patients had a monoclonal gammopathy associated with cryofibrinogenemia. In some reports, the M protein was demonstrated to cause the precipitation of cryofibrinogen in a healthy control, further supporting its pathogenic role in the disease [9,10]. HBV/HCV infection alone did not emerge as a significant driver of cryofibrinogenemia in this case series report: 3/50 patients were found to be positive to hepatotropic virus serology. Eighteen patients showed significant autoimmune comorbidities, with arthritis being the most represented symptom after Raynaud’s phenomenon, which in itself showed great overlap with cryoprotein-related signs and symptoms. Overlap with cryoglobulinemia was marginal in this series, with three patients reportedly having tested positive for both cryoglobulinemia and cryofibrinogemia. Skin or muscle biopsies were performed in 34/50 cases, with polarizing results: 13/34 had varying degrees of inflammation, with leukocytoclastic vasculitis being the most common pattern reported, while 21/34 cases exhibited a uniquely thrombotic angiopathic histology with no signs of inflammation. In patients with cutaneous leukocitoclastic vasculitis findings, renal involvement was reported in six cases, three of which were severe. In the 21 patients with no signs of cutaneous inflammation, kidney involvement was reported in three cases. Whether patients with inflammatory histology at skin biopsy have worse renal outcomes remains to be further verified with larger population samples. Out of the 15 total patients with variable kidney involvement, a biopsy was performed in 10: a membranoproliferative pattern was reported in 8 patients, 1 had AA amyloidosis, and 1 had normal findings. The treatment strategies employed in this case series were broad: the most prescribed drugs, in primary forms, were steroids, colchicine, aspirin, and anticoagulants. The secondary forms of prescriptions were similar. The immunochemistry detection of subclass 3 IgG/k in the glomerular deposits of the third biopsy of our patient was intriguing. Fibrinogen cold precipitation can be directly related to an IgG-k monoclonal paraprotein [9] and monoclonal anti-fibrinogen antibodies were first described three decades ago [11]. See Figure 3 for the detailed search strategy.

## 5. Discussion

What was especially surprising in our case was the unexpected reappearance in the serum of clonally restricted immunoglobulins (identified only once at the very onset of the disease, in trace amounts, and persistently negative during the subsequent follow-up), which was coincident with the identification of monotypic deposits of IgG1-k in the third renal biopsy. We suspect that this reappearing molecular component could acquire an anti-fibrinogen reactivity and that its increase in titers marked a critical turning point in the clinical history of our patient. Indeed, it was coincident with the loss of responsiveness to the procedures of fibrinogen removal from circulation alone. To explore the possibility that this emerging clonally restricted IgG could have triggered the rapid and severe progression of the disease, a series of laboratory investigations were carried out.

Briefly, we conducted tests with the patient’s serum (which, after clotting, did not include fibrinogen or clotting factors but included immunoglobulins and other proteins) and plasma from a pool of healthy donors.

After 1 week of incubation and per-protocol centrifugation, no cryoprecipitate was identified in the serum of the patient, in healthy donor plasma pool samples, or in a healthy control serum sample when mixed with pooled donor plasma. In contrast, when the patient’s serum was mixed with plasma from a healthy donor pool and was incubated at 4°c for 1 week in Wintrobe tubes, after the per-protocol centrifugation, a significant cryoprecipitate (ranging from 5 to 20%) could be identified in 10 separate tests, definitely indicating a potential pathogenic role of the monoclonal gammopathy in our case.

We also evaluated whether a dose-dependent correlation could be demonstrated, mixing 25–50–75% of patient serum to corresponding amounts of pooled donor plasma. Maximum cryoprecipitate formation was achieved in the 50% plasma + 50% serum, amounting to about 20%.

This case offers significant insights into cryofibrinogen-associated kidney disease, particularly highlighting the complex interplay between monoclonal gammopathy and established cryofibrinogenemia. The evidence of cryoprecipitation observed when combining the patient’s serum with healthy donor plasma strongly suggests a causal relationship between the monoclonal gammopathy and cryofibrinogenemia. This finding aligns with emerging perspectives that some forms of cryofibrinogenemia may indeed represent a variant of monoclonal gammopathy of renal significance (MGRS). Recognizing this connection is crucial, especially when renal involvement occurs without the systemic manifestations of cryoproteinemia.

The patient’s renal disease was primarily driven by cryofibrinogen, as evidenced by the positive response to plasma therapy. This reinforces the idea that cryofibrinogenemia can present primarily as a renal disorder, even in the absence of other systemic symptoms. It underscores the importance of considering this diagnosis in patients with unexplained renal impairment, especially when traditional biomarkers do not fully capture the disease activity.

The role of the kidney biopsy and electron microscopy examination was paramount in this case. Conventional biomarkers were irrelevant, making biopsy a critical tool for diagnosis and treatment decision-making. The initial biopsy provided definitive evidence of cryofibrinogen deposits and renal involvement. The utility of repeat biopsies for an ongoing assessment of the kidney’s histological changes is evident. Biopsy-driven management likely delayed the need for renal replacement therapy by enabling timely and appropriate adjustments to the patient’s treatment regimen.

In managing relapsing–remitting diseases like this one, the timing of therapeutic interventions is crucial. Initially, the patient did not respond to high-dose steroid therapy, leading to the implementation of plasmapheresis. This treatment proved to be highly effective, rapidly clearing the pathogenic cryoprotein and stabilizing the patient’s condition, even during advanced stages of the disease. The success of plasmapheresis in this context highlights its importance as a therapeutic option, particularly when swift action is necessary to prevent further renal damage.

In conclusion, this case underscores the complexity and challenges of managing cryofibrinogen-associated kidney disease. The integration of personalized management strategies, such as the use of kidney biopsies and targeted therapies like plasmapheresis, has been instrumental in improving patient outcomes. As this condition is rare, continued documentation and study are essential to further our understanding and refine treatment approaches for similar cases.

## Data Availability

The original contributions presented in this study are included in the article. Further inquiries can be directed to the corresponding author.
